# More than Five Decades of Proton Therapy: A Bibliometric Overview of the Scientific Literature

**DOI:** 10.3390/cancers15235545

**Published:** 2023-11-23

**Authors:** Maria Giulia Vincini, Mattia Zaffaroni, Marco Schwarz, Giulia Marvaso, Federico Mastroleo, Stefania Volpe, Luca Bergamaschi, Giovanni Carlo Mazzola, Giulia Corrao, Roberto Orecchia, Barbara Alicja Jereczek-Fossa, Daniela Alterio

**Affiliations:** 1 Division of Radiation Oncology, IEO European Institute of Oncology IRCCS, 20141 Milan, Italy; mariagiulia.vincini@ieo.it (M.G.V.); federico.mastroleo@ieo.it (F.M.); stefania.volpe@ieo.it (S.V.); giulia.corrao@ieo.it (G.C.); barbara.jereczek@ieo.it (B.A.J.-F.);; 2 Radiation Oncology Department, University of Washington, Seattle, WA 98109, USA; marcosch@uw.edu; 3 Department of Translational Medicine, University of Piemonte Orientale, 28100 Novara, Italy; 4 Scientific Directorate, IEO European Institute of Oncology IRCCS, 20141 Milan, Italy; roberto.orecchia@ieo.it; 5 Department of Oncology and Hemato-Oncology, University of Milan, 20122 Milan, Italy

**Keywords:** proton therapy, bibliometric analysis, bibliometrics

## Abstract

**Simple Summary:**

The therapeutic potential of proton therapy (PT) was first recognized in 1946 by Robert Wilson, and nowadays, over 100 proton centers are in operation worldwide, and more than 60 are under construction or planned. Bibliometrics allows us to assess the growth and development of the field and provides an overview of the most relevant sources, authors, and institutions in the chosen field. Additionally, keyword analysis can be performed to provide details that could aid in identifying research trends and hot topics. Indeed, bibliometrics allows for identifying the basic and dominant themes and trends, as well as those emerging or declining. The aim of this study is to provide a comprehensive bibliometric analysis of the current status and trends in scientific literature in the PT field. The analysis will give an overview of past scientific production and, most importantly, provide a useful point of view on the future directions of the research activities.

**Abstract:**

Background: The therapeutic potential of proton therapy (PT) was first recognized in 1946 by Robert Wilson, and nowadays, over 100 proton centers are in operation worldwide, and more than 60 are under construction or planned. Bibliometric data can be used to perform a structured analysis of large amounts of scientific data to provide new insights, e.g., to assess the growth and development of the field and to identify research trends and hot topics. The aim of this study is to provide a comprehensive bibliometric analysis of the current status and trends in scientific literature in the PT field. Methods: The literature on PT until the 31st December 2022 in the Scopus database was searched, including the following keywords: *proton* AND *radiotherapy* AND *cancer*/*tumor* in title, abstract, and/or keywords. The open-source R Studio’s Bibliometrix package and Biblioshiny software (version 2.0) were used to perform the analysis. Results: A total of 7335 documents, mainly articles (n = 4794, 65%) and reviews (n = 1527, 21%), were collected from 1946 to 2022 from 1054 sources and 21,696 authors. Of these, roughly 84% (n = 6167) were produced in the last 15 years (2008–2022), in which the mean annual growth rate was 13%. Considering the corresponding author’s country, 79 countries contributed to the literature; the USA was the top contributor, with 2765 (38%) documents, of whom 84% were single-country publications (SCP), followed by Germany and Japan, with 535 and 531 documents of whom 66% and 93% were SCP. Considering the themes subanalysis (2002–2022), a total of 7192 documents were analyzed; among all keywords used by authors, the top three were radiotherapy (n = 1394, 21% of documents), intensity-modulated radiotherapy (n = 301, 5%), and prostate cancer (n = 301, 5%). Among disease types, prostate cancer is followed by chordoma, head and neck, and breast cancer. The change in trend themes demonstrated the fast evolution of hotspots in PT; among the most recent trends, the appearance of flash, radiomics, *relative biological effectiveness (RBE)*, and *linear energy transfer (LET)* deserve to be highlighted. Conclusions: The results of the present bibliometric analysis showed that PT is an active and rapidly increasing field of research. Themes of the published works encompass the main aspects of its application in clinical practice, such as the comparison with the actual photon-based standard of care technique and the continuing technological advances. This analysis gives an overview of past scientific production and, most importantly, provides a useful point of view on the future directions of the research activities.

## 1. Introduction

The therapeutic potential of proton beams was first recognized in 1946 by Robert Wilson, with the idea that a proton beam can provide a higher energy deposit in tumors by minimizing radiation exposure to surrounding healthy organs [1]. This hypothesis was developed by the Lawrence Berkeley Laboratory, which initiated research related to proton irradiation in the clinical setting of patients with malignant tumors. The first patient was treated in 1954 [2] with a synchrocyclotron at the University of California, Berkley. The first hospital-based proton therapy (PT) facility was established in 1990 at the Loma Linda University Medical Center (CA), and since then, PT experienced exponential growth in both facilities available for clinical use and the number of treated patients [3]. Nowadays, over 100 proton centers are in operation worldwide, and more than 60 are under construction or planned for a total of more than 311,000 patients treated with protons by the end of 2022 [4].

PT is associated with several clinical, physical, and translational aspects. For instance, the peculiar dose distribution of protons leads to a significant reduction in the low “dose bath” absorbed by surrounding healthy tissues that are typically seen in photon-based techniques like intensity-modulated radiotherapy (IMRT). This could result in several advantages, like reducing (1) acute and late treatment-related side effects, (2) radiation-induced secondary-cancer risk (mainly in young patients), and (3) immune system depression (thanks to the lower exposure of circulating lymphocytes and immune organs like lymph nodes) [5,6,7,8,9,10,11,12]. Moreover, technological advances have allowed the transition from passive scattering to pencil-beam scanning (PBS), with even higher conformity of the dose distribution—a technique routinely introduced by the Paul Scherrer Institute (Villigen, Switzerland) in the late 1990s. Whether this improvement could translate into a better cost/benefit ratio in clinical results is still a matter of investigation [13,14]. To date, a number of issues remain to be addressed in PT, such as the dosimetric impact of uncertainties on the delivered dose distributions versus the desired one and the criteria to define clinical indications in a scenario of limited resources. Therefore, there is consensus on the need to improve scientific evidence about PT from the bench to the bedside [15].

Bibliometric data can be used to perform a structured analysis for large amounts of scientific data using various metrics, which comprise, for instance, the number of publications over time, citation counts, institutional affiliations, authors, and countries’ contributions. Bibliometrics allows us to assess the growth and development of the field and provides an overview of the most relevant sources, authors, and institutions in the chosen field. Additionally, keyword analysis can be performed to provide details that could aid in identifying research trends and hot topics. Indeed, bibliometrics allows for identifying the basic and dominant themes and trends, as well as those emerging or declining. In the past, several bibliometric analyses have been carried out in various areas and, recently, also in the radiation oncology field [16,17,18,19,20,21].

Up to today, no bibliometric analysis regarding PT has been performed yet; therefore, in this study, we aim to provide a comprehensive analysis of the status of the available scientific documents in the field of PT. This will allow us to have a snapshot of current topics and developing trends from 1947, the year of the first publication on the matter, to 2022.

## 2. Material and Methods

All English language documents available in the Scopus database were considered eligible for the analysis.

The search string used was the following: (TITLE-ABS-KEY ((proton) AND radiotherapy) AND (TITLE-ABS-KEY (cancer OR tumor)) AND NOT (TITLE-ABS-KEY (pump))) AND PUBYEAR < 2023. All documents up to the date of the research (16 June 2023) were considered.

Data were exported in BibTex format, comprehensive of all the metadata regarding the title, abstract, citation information, corresponding author’s country, bibliographical information, authors’ keywords, and cited references in the manuscript. The Bibliometrix R package in the R environment (R-Studio 0.98.1091 software) and the Biblioshiny (version 2.0) [22] interface were used for the analysis.

The entire sample of documents was used for analyses regarding scientific production over the years. Based on the assumption of a uniform contribution of all co-authors to each document and neglecting the different types of documents, top relevant sources and authors were assessed based on the number of documents. Affiliations of all authors were considered to assess the top relevant institutions, and the corresponding author’s country was considered to assess the top productive countries and network collaboration regarding countries. Country production was also evaluated through a productivity index, defined as the number of documents divided by the number of PT centers in operation at the time of the study. Moreover, single-country publications (SCP) and multiple-country publications (MCP), namely documents with authors from one country and documents in which at least one of the co-authors had a different country affiliation compared to the corresponding author, respectively, were considered as another index of collaborations among nations.

Given the wide time span involving scientific production and the fast pace of development, analyses of keywords and trends focused on the 2002–2022 period only to obtain a glimpse of the major driving themes of the last two decades. The authors’ keywords analysis included frequent PT-associated keywords and a keywords co-occurrence network. A list of synonyms was loaded and is provided in the Supplementary Materials Table S1. In addition, to see the evolution of trend topics over time, the top three keywords for each year were considered. Lastly, the thematic map [23] was produced to provide the relevant lines of research.

## 3. Results

### 3.1. Overview

A total of 7335 documents were collected from 1947, the year of the first paper on the clinical application of PT in Scopus, to 2023, from 1054 sources. Roughly 84% (n = 6167) of the documents were produced during the last 15 years (2008–2022), and the mean annual growth rate in this period was 13%. The retrieved documents were mainly articles (n = 4794, 65%) and reviews (n = 1527, 21%).

### 3.2. Sources and Authors

**Figure 1:**
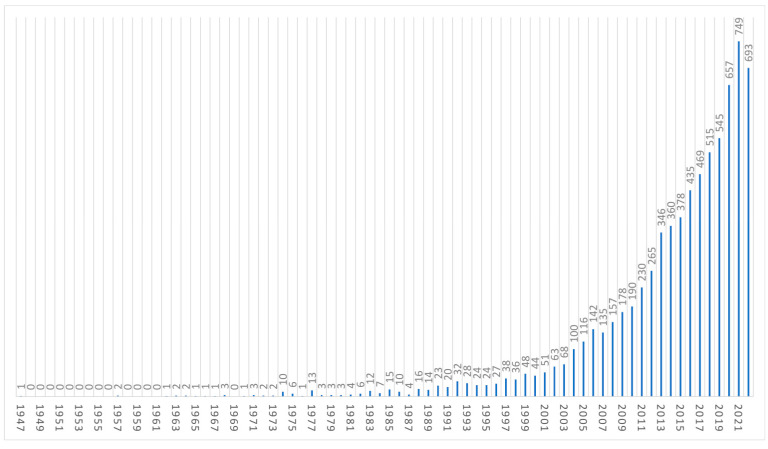
Annual scientific production in terms of documents produced.

A total of 21,696 authors contributed to the literature, and on average, each document was written by seven co-authors. The majority of contributors (n = 14,436, 70%) were occasional authors who contributed with one document only, while the top 10 authors contributed to 985 documents, 13% of the total. The last three completed years (years 2020–2021–2022) were the most productive, with 657, 749, and 693 documents, respectively (Figure 1). The top ten relevant sources and affiliations are provided in the Supplementary Materials Table S2. The top sources were the *International Journal of Radiation Oncology, Biology and Physics* (n = 727 documents), *Radiotherapy and Oncology (n = 375 documents),* and *Physics In Medicine and Biology* (n = 298 documents).

The top three affiliations in terms of publication volume were the Massachusetts General Hospital—Harvard Medical School (Boston, MA, USA, n = 1050), the University Of Texas MD Anderson Cancer Center (Houston, Texas, USA, n = 646), and the Mayo Clinic (Rochester, MN, USA, n = 391 documents).

### 3.3. Countries

**Figure 2:**
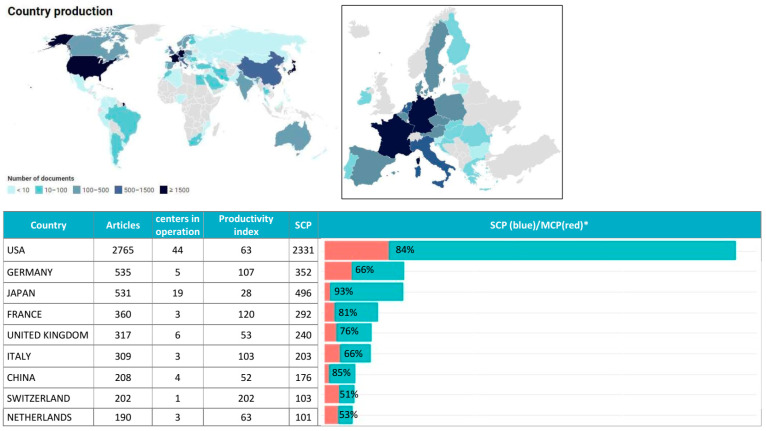
(**Top**): Country-specific production worldwide (left) and a focus on Europe (right) considering the corresponding author’s country; (**Bottom**): top fifteen most productive countries considering the corresponding author’s country and proportion between single-country publications (SCP) and multiple-country publications (MCP). * Publications with authors from more than one country.

A total of 79 countries contributed to the literature (Figure 2, top). Among all considered documents, 21.1% were publications with authors from at least two countries.

**Figure 3:**
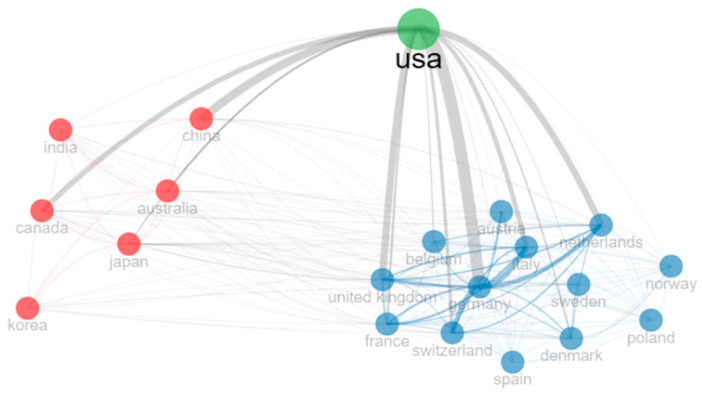
Countries collaboration network.

Considering the corresponding author’s country, the USA was the top contributor, with 2765 (38%) documents, of whom 84% were SCP, followed by Germany and Japan, with 535 and 531 documents, of whom 66% and 93% were SCP (Figure 2, bottom). Switzerland, France, and Germany were the countries with the greater productivity indexes. The country collaboration network (Figure 3) showed the main node, the USA, on its own, was strongly linked to two clusters: the blue one with European countries, with the strongest links with Germany and the United Kingdom, and the red one, with the strongest links with Canada and China.

### 3.4. Themes and Trends

Considering the themes’ subanalysis (2002–2022), a total of 7192 documents were analyzed. Table 1 shows the top 20 PT-associated keywords, with the top 3 being *radiotherapy* (n = 1394, 21% of documents), *intensity-modulated radiotherapy* (n = 301, 5%), and *prostate cancer* (n = 301, 5%). Among the disease types, prostate cancer is followed by chordoma, head and neck, and breast cancer.

**Figure 4:**
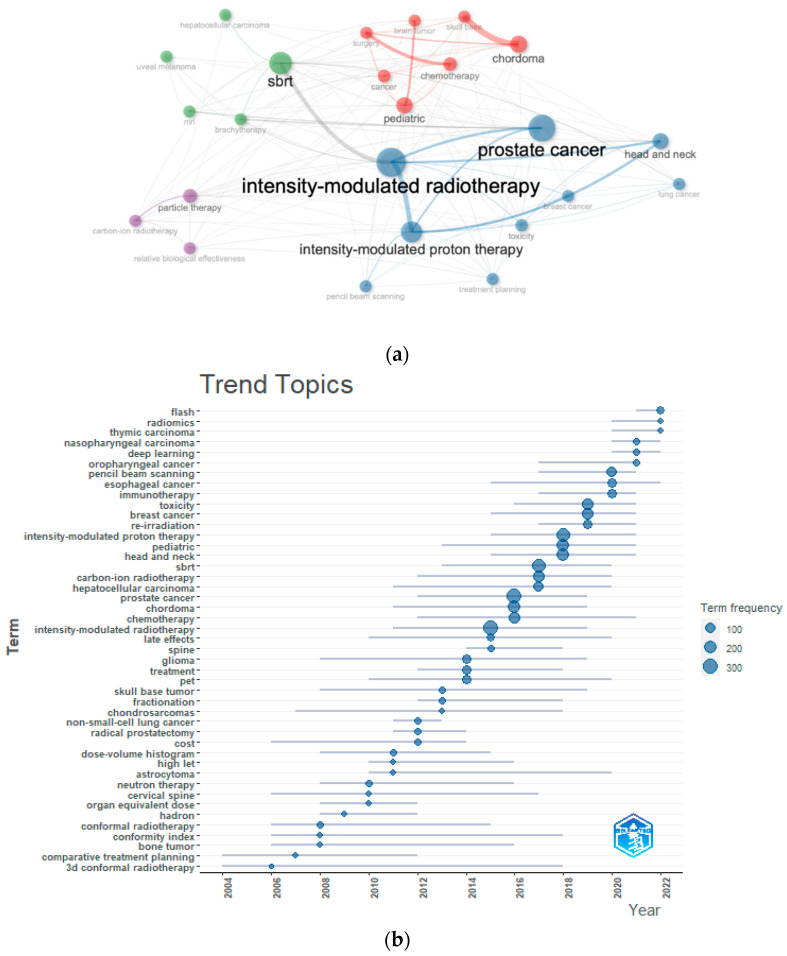
(**a**) Keywords co-occurrence network. Each vertex represents a keyword, and its size is proportional to the occurrence. Each cluster (colors) can be considered as a topic macro-area. The proximity among keywords in the network indicates a large proportion of articles addressing them together. “Radiotherapy” was excluded from the figure because it would have covered the other keywords. List of abbreviations: SBRT (stereotactic body radiation therapy). (**b**) Thematic evolution of keywords (2002–2022); blue dots: top three proton therapy-associated keywords for each year; the line-span indicates the interquartile range of the keyword distribution over years.

The co-occurrence network among the 25 top PT-associated keywords (Figure 4a) showed four different clusters. Among them, the wider cluster (blue), within *intensity-modulated radiotherapy, intensity-modulated PT*, and *prostate cancer* stand out; then, this is followed by the *pediatric/intracranic tumor* cluster (red), the stereotactic body radiation therapy (SBRT) cluster (green), and lastly, a cluster constituted by *particle therapy*, *relative biological effectiveness*, and *carbon-ion radiotherapy.* Among different cancer subsites in adults, prostate, head and neck, and base of the skull were the most frequently cited tumors. Moreover, the field of pediatrics was well-represented.

Figure 4b shows the change in trend themes over the period 2002–2022, with the three most frequent keywords for each year. The most recent topics were flash therapy and radiomics, which appeared in 2021 and 2020 and were in the top three keywords in 2022. From a clinical point of view, several tumors have been represented in the literature during the last twenty years and were mainly prostate, brain, and head and neck tumors in pediatric and lung settings.

**Figure 5:**
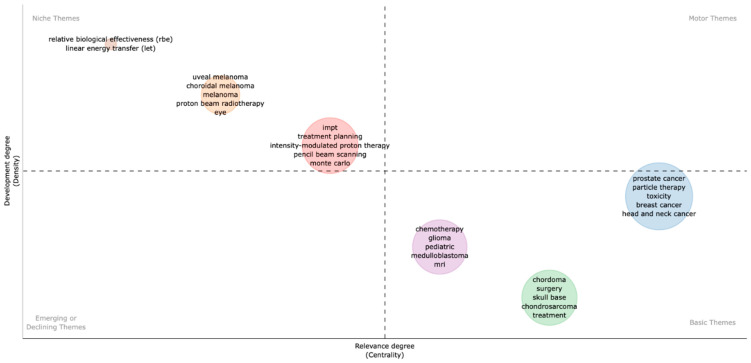
Thematic map [23] highlights the significant modifications in authors’ keywords (2002–2022). Research themes are divided into four categories: (1) “Motor themes”, with a high degree of centrality and density, refer to topics within the field that have a high level of importance and are continually developing; (2) “Niche themes”, highly developed but isolated, refer to topics that are strongly developed but still marginal for the domain under investigation; (3) “Emerging or declining themes”, with a low level of centrality and density, refer to themes that are not fully developed or marginally interesting for the domain; (4) “Basic themes”, with high centrality but low development, refer to transversal themes that are considered to be fundamental and cross-cutting but have a lower level of development.

The thematic map can be seen in Figure 5. This graph allows the identification of the hot topics (higher values of centrality and density) in the upper-right quadrant, the basic topics (higher values of centrality and lower values of density) in the lower-right quadrant, the peripheral topics (lower values of centrality and density) in the lower-left quadrant, and the niche topics (lower values of centrality and higher values of density). The map highlights *RBE* and linear energy transfer (*LET)* as niche themes, isolated topics at a very high degree of development (upper-left side); less isolated and at a relevant degree of development were topics related to eye disease (*uveal melanoma, choroidal melanoma, eye*). *IMPT* and treatment planning-related themes (*treatment planning, PBS, Monte Carlo*) were central topics, and keywords, such as *chordoma, surgery, skull-base, condrosarcoma, chemotherapy, glioma, pediatric,* and *medulloblastoma* represent basic and transversal themes, and a low degree of development. Lastly, *prostate cancer, particle therapy, toxicity, breast cancer, and head and neck cancer* were basic themes at an average degree of development.

## 4. Discussion

Our bibliometric analysis included more than 7300 documents and allowed us to assess the state-of-the-art and the evolution of the current literature in the field of PT [24]. PT has undergone exponential growth in terms of treating facilities and treated patients. Such growth is also reflected by the number of scientific publications. Not surprisingly, we can observe that the scientific output has been increasing steadily, reaching more than 600 articles in each of the last three years (2020, 2021, and 2022).

### 4.1. Sources and Authors

The majority of top-contributor journals were addressed to the radiation oncologists and medical physics scientific communities. The two top sources, known in the field as the *Red Journal* and the *Green Journal*, are official journals of international organizations in the field of radiation oncology, namely the American Society for Radiation Oncology (ASTRO) and European Society for Radiotherapy and Oncology (ESTRO), respectively. They cover almost all areas of interest relating to the clinical application of radiation oncology. The finding that the majority of papers have been published in these journals represents a guarantee of the robustness of the methodology and reliability of the results reported in the published works. Moreover, it also reflects the high interest in the radiation oncology scientific community on the topic.

The third journal, *Physics in Medicine and Biology,* refers to the Institute of Physics and Engineering in Medicine (IPEM), whose mission is to improve health through physics and engineering in medicine, pointing at the inescapable link between PT and the development of technological and physical knowledge. Indeed, technical aspects are of paramount importance for translating the more favorable physical characteristics of protons into safe and effective treatments. In the last decades, several aspects of PT technological assets have been investigated, such as the implementation of the pencil-beam scanning (PBS) technique, the refinement of image guidance, set-up strategy techniques, as well as the development of compact systems [25,26,27,28]. Indeed, PBS and intensity-modulated PT (IMPT) were present as the top-associated keywords in the period of 2017–2021 [15,27]. The authors’ indexing is naturally related to the publication type, and we found an equal distribution of physicians and physicists among the top four contributors.

### 4.2. Countries

Regarding the countries and affiliations analysis, the USA was found to be the top country contributor in terms of volume of publications, and eight out of the ten top affiliations are from facilities in the USA.

Particularly, the first top affiliation was the Massachusetts General Hospital (MGH)—Harvard Medical School (Boston, MA, USA), one of the leading hospitals in the USA. Indeed, one of the pioneers of PT was the research facility at the Harvard Cyclotron Laboratory (HCL) in Cambridge, Massachusetts, operating in conjunction with the MGH. Patient treatment lasted from 1961 to 2002, which was when the clinical program was transferred to the Northeast Proton Therapy Center (NPTC) at MGH [29]. MGH was thus one of the first hospitals in the world to establish its own proton therapy for cancer and, by now, has treated more than 10,000 patients [30].

Despite the huge production of documents, the USA productivity index is among the lowest. Indeed, the USA is the country with the highest number of PT facilities, with a total of 44 in operation and more than 10 under construction/planning stages [31]. It is followed by Japan, with 19 PT facilities in operation and 2 under construction, which, in turn, resulted in being second in terms of article production. Moreover, the USA, up to December 2021, has treated approximately 143,000 patients—51% of all patients treated worldwide at that date—and is, by de facto, the leading country in PT [4]. Moreover, the country collaboration network (Figure 3) highlighted the USA as an important node and showed a tendency for European countries to collaborate between them (blue cluster).

Scientific production in the United States is certainly also favored by the presence of many PT registries. A total of ten monocentric and multicentric PT registries are actively recruiting patients, and among them, the Proton Collaborative Group (PCG) registry (NCT01255748) is currently collecting data from 25 proton facilities [32].

Similarly, collaboration among European countries is boosted by initiatives promoted by Scientific Societies. A collaboration between the European Organization for Research and Treatment of Cancer (EORTC) and the ESTRO resulted in the creation of ParticleCare (EORTC 1833-RP), a common research platform to collect real-world data of patients treated with protons and C-ions in European centers [33].

Another project currently developing an infrastructure for automatic data registration is the PROTRAIT (PROton Therapy ReseArch regIsTry) [34] initiative in the Netherlands. The specific aim of the project is to establish an infrastructure for automatic data registration related to novel PT in the Netherlands to validate and strengthen the model-based approach used in this Country.

It should be noted that Switzerland is the country with the highest productivity index. This is not surprising, given its central role in the history of proton delivery, which can explain why such a small country with a small population and only one PT center is the eighth most productive country in terms of publications and the one with the highest productivity index.

In this scenario, low and middle-income countries (LMIC) remain very poorly represented. A study by Xia et al. [35] assessed extremely high inequality in the availability of PT centers throughout the world. Particularly in LMIC, cancer patients often have no access to PT despite the fact that these nations have a higher population and more cancer patients [35]. Undoubtedly, the scarce production of literature is another index of disparities affecting these nations.

### 4.3. Themes and Trend

The keywords subanalysis over the last twenty years reveals significant heterogeneity in the clinical keywords. This finding may be due to different clinical indications of PT among different countries. Zientara et al. [36] carried out a scoping review to explore various national and international clinical decision-making tools and dose comparison methods used for selecting cancer patients for proton versus X-ray radiation therapy. Great variability in indications emerged across different countries, especially for adult patients. With the exception of pediatric patients and adult patients with base of skull and chondrosarcomas [36], which were basic and transversal themes in the thematic map, there is uncertainty regarding patient cohorts that may benefit most from PT.

IMRT emerges from the associated keywords. Nowadays, IMRT and VMAT represent the standard of care for the vast majority of oncologic disease candidates for radiation treatment [37]. The methodology used does not allow us to assess the number of dosimetric studies emerging from the search. However, the strict association of IMRT with PT detected in the present analysis might reflect the high production of treatment planning studies in the last few years.

Among the technological advances in PT, FLASH therapy was found to be among the most recent and active fields of research in 2022. FLASH therapy allows treatment to be delivered at very high dose rates, and this approach has been proposed as a possible new frontier for radiation treatment, stimulating interest in preclinical, clinical, and technological studies [38,39,40]. Thus far, 10 patients have been treated within the FAST-01 non-randomized trial, the first and only trial on flash with protons. This trial was carried out at Cincinnati Children’s/UC Health Therapy Center for patients with bone metastases in arms and legs, receiving a single palliative treatment fraction of 8 Gy [41,42].

Radiomics refers to the extraction of quantitative mineable data (*features*) from medical images and has been applied within oncology to enhance diagnosis, prognosis, and clinical decision support to provide increasingly tailored treatment [43,44,45]. The presence of radiomics, together with *deep learning*, found within the top keywords in 2021 and as a theme with a high degree of development, is not surprising; radiomics has experienced strong growth in recent years in the field of oncology, thanks to the ever-rising availability of images and technological advances in machine learning and artificial intelligence.

RBE and LET were also found to be topics at a high development rate in the thematic map. Currently, an RBE value of 1.1 is used to determine the clinical proton doses, which is thus 10% reduced due to the expected enhanced effectiveness [46]. However, this value represents only an approximation in the mid-part of the spread-out Bragg peak (SOBP). The use of a constant is currently under investigation, as RBE, in principle, may change as a function of both the proton LET and the specific clinical endpoint under consideration [47,48]. Radiobiological research on RBE is ongoing so as to fully comprehend how to model the complex relationship between RBE and LET in the SOBP; for example, the work package number six of the European Particle Therapy Network (EPTN) is fully centered on radiobiology and RBE [48,49].

The analysis performed in this work can be easily reproduced thanks to the Bibliometrix package [22], which is freely available to anyone and can be easily used in the R studio environment. The main limitation is that the literature search included only the Scopus database, which may have excluded other published work and citations. Moreover, the package used does not allow the documents collected to be divided into, e.g., mainly clinical papers, papers reporting trial results, methodological papers, or mainly physical papers. Lastly, the lack of bibliometric analysis regarding RT approaches comparable to PT (e.g., SBRT, FLASH) prevents the eventual comparison of results that could better highlight the specificities of PT.

For instance, assessing the number of papers reporting trial results by site of disease could be an interesting feature to add and would provide extra information. Although the theoretical benefit of protons over photons is high, to date, clinical shreds of evidence supporting its use are still conflicting [50]. As a matter of fact, PT can be considered an established treatment approach, with more than 100 facilities worldwide treating approximately 400 patients per year [31]. Promising results have been reported for several types of cancers, mainly in terms of toxicity reduction. Nonetheless, the majority of the reported data were retrieved from small, non-randomized studies, and widespread discussion regarding the lack of evidence of superior benefits makes PT a technique that still needs to find a role in radiation oncology [15].

Despite the limitations, we believe that the present analysis represents a reliable snapshot of the literature concerning PT.

## 5. Conclusions

PT is an active and rapidly increasing field of research and clinical application. Themes of the published works encompass the main aspects of its application in clinical practice, such as the comparison with the actual photon-based standard of care technique and the continuing technological advances. This analysis gives an overview of past scientific productions and, most importantly, provides a useful point of view on the future directions of research activities.

## Figures and Tables

**Table 1:** Top 20 PT-associated keywords.

Words	Occurrences
Radiotherapy	1394
Intensity-modulated radiotherapy	301
Prostate cancer	301
Stereotactic body radiation therapy	255
Intensity-modulated proton therapy	219
Chordoma	182
Pediatric	175
Head and neck	174
Particle therapy	157
Chemotherapy	145
Cancer	137
Toxicity	131
Breast cancer	124
Carbon-ion radiotherapy	122
Surgery	120
Lung cancer	119
Brain tumor	110
Pencil-beam scanning	110
Hepatocellular carcinoma	105
Relative biological effectiveness	97

## Data Availability

Data supporting the results of this study are available from the corresponding authors upon reasonable request.

## Supplementary Materials

The following supporting information can be downloaded at: https://www.mdpi.com/article/10.3390/cancers15235545/s1, Table S1: Considered synonyms in the present analysis. Table S2: Top ten sources and affiliations.
