# Supplementary material for: More than Five Decades of Proton Therapy: A Bibliometric Overview of the Scientific Literature
Source: Cancers (Basel). 2023 Nov 23;15(23):5545. doi: 10.3390/cancers15235545 (PMC10705139; doi:10.3390/cancers15235545)
Supplement: Supplementary file 1 [file cancers-15-05545-s001.zip › cancers-2696963-supplementary.pdf]

Supplementary Materials: More than Five Decades of Proton Therapy: A Bibliometric Overview of the Scientific Literature

Maria Giulia Vincini [1], Mattia Zaffaroni [1,*], Marco Schwarz [2], Giulia Marvaso [1,*], Federico Mastroleo [1,3], Stefania Volpe [1], Luca Bergamaschi [1], Giovanni Carlo Mazzola [1], Giulia Corrao [1], Roberto Orecchia [4], Barbara Alicja Jereczek-Fossa [1,5] and Daniela Alterio [1]

1 Division of Radiation Oncology, IEO European Institute of Oncology IRCCS, 20141 Milan, Italy
2 Radiation Oncology Department, University of Washington - Fred Hutchinson Cancer Center, Seattle, WA 98109, USA
3 Department of Translational Medicine, University of Piemonte Orientale, 28100 Novara, Italy
4 Scientific directorate, IEO European Institute of Oncology IRCCS, 20141 Milan, Italy
5 Department of Oncology and Hemato-Oncology, University of Milan, 20122 Milan, Italy
* Correspondence: Email: Mattia.zaffaroni@ieo.it

Table S1. – considered synonyms in the present analysis.

**adjuvant radiation; adjuvant; adjuvant radiotherapy**
bnct; boron neutron capture therapy
brain tumor; brain tumors; brain tumour; brain tumours
cancer; tumor
carbon-ion radiotherapy; carbon; carbon ion; carbon ions; carbon-ion therapy
cardiac toxicity; cardiotoxicity
charged particle therapy; charged particles; charged-particle therapy
chemoradiation; chemoradiotherapy
chordoma; chordomas
clinical trial; clinical trials
flash; flash radiotherapy
head and neck; head and neck cancer; head and neck neoplasms
heavy ion radiotherapy; heavy ions; heavy ion radiotherapy; ion beam therapy; ion therapy
hypofractionation; hypofractionated
igrt; image guidance; image-guided radiation therapy; image-guided radiotherapy
intensity modulation; intensity-modulated
intensity-modulated proton therapy; impt; intensity modulated proton therapy; intensity-modulated proton therapy; intensity-modulated proton therapy (impt)
intensity-modulated radiotherapy; imrt; intensity-modulated radiation therapy; intensity modulated radiation therapy; intensity modulated radiotherapy
let; linear energy transfer; linear energy transfer (let)
local control; local tumor control
metastasis; metastases
mri; magnetic resonance; magnetic resonance imaging
neutron therapy; neutrons
non-small cell lung cancer; nsclc
particle radiotherapy; particle beam therapy
pediatric; children; child; childhood; childhood cancer; paediatric
pencil beam scanning; pbs; pencil beam; pencil-beam scanning; proton pencil beam scanning
pet; pet/ct; positron emission tomography
photon radiotherapy; photon; photon therapy; photons
prostate cancer; prostate; prostatic neoplasms
proton therapy; proton beam therapy; proton radiotherapy; proton beam; proton radiation; proton beam irradiation; proton beam radiation; proton beam radiation therapy; proton beam radiotherapy; proton beam therapy (pbt); proton beams; proton irradiation; protontherapy; proton; protons; proton radiation therapy; proton-beam therapy
radiotherapy; radiation therapy; radiation; radiation oncology
re-irradiation; reirradiation
relative biological effectiveness; relative biological effectiveness (rbe); rbe

sbrt; stereotactic radiotherapy; stereotactic radiosurgery; stereotactic radiosurgery; stereotactic body radiotherapy; radiosurgery; stereotactic; stereotactic body radiation therapy; stereotactic body radiation therapy (sbrt)
skull base tumor; skull base tumors
surgery; resection
survival; survivorship
toxicity; toxicities
tumor recurrence; recurrence
vmat; volumetric modulated arc therapy

Table S2. - top ten sources and affiliations.

Source		Number of documents
**INTERNATIONAL JOURNAL OF RADIATION ONCOLOGY BIOLOGY PHYSICS**		727
RADIOTHERAPY AND ONCOLOGY		375
PHYSICS IN MEDICINE AND BIOLOGY		298
MEDICAL PHYSICS		260
ACTA ONCOLOGICA		225
RADIATION ONCOLOGY		161
FRONTIERS IN ONCOLOGY		157
CANCERS		148
BRITISH JOURNAL OF RADIOLOGY		106
CLINICAL ONCOLOGY		91
**Affiliation**		**Articles**
MASSACHUSETTS GENERAL HOSPITAL - HARVARD MEDICAL SCHOOL	Boston, MA, USA	1050
THE UNIVERSITY OF TEXAS MD ANDERSON CANCER CENTER	Houston, TX, USA	646
MAYO CLINIC	Rochester, MN, USA	391
UNIVERSITY OF TSUKUBA	Tsukuba, Japan	310
UNIVERSITY OF PENNSYLVANIA	Philadelphia, PA, USA	274
MEMORIAL SLOAN KETTERING CANCER CENTER	New York, NY, USA	228
UNIVERSITY OF CALIFORNIA	California, USA	198
UNIVERSITY OF FLORIDA	Gainesville, FL, USA	190
UNIVERSITY OF TEXAS	Austin, TX, USA	184
INSTITUT CURIE	Paris, FR	176
